# Relationship between periodontitis and systemic health conditions: a narrative review

**DOI:** 10.12771/emj.2025.00101

**Published:** 2025-04-14

**Authors:** Min-Young Kim, Eun-Kyoung Pang

**Affiliations:** 1 College of Medicine, Ewha Womans University, Seoul, Korea; 2 Department of Periodontology, College of Medicine, Ewha Womans University, Seoul, Korea

**Keywords:** Cardiovascular diseases, Diabetes mellitus, Obesity, Oral health, Periodontitis

## Abstract

This review examines the bidirectional relationship between periodontitis and systemic health conditions, offering an integrated perspective based on current evidence. It synthesizes epidemiological data, biological mechanisms, and clinical implications to support collaborative care strategies recognizing oral health as a key component of overall wellness. Periodontitis affects 7.4% to 11.2% of adults worldwide, and its prevalence increases with age. Beyond its local effects, including gingival inflammation, periodontal pocket formation, and alveolar bone loss, periodontitis is associated with various systemic conditions. Emerging evidence has established links with obesity, diabetes mellitus, cardiovascular disease, chronic kidney disease, inflammatory bowel disease, rheumatoid arthritis, respiratory diseases, adverse pregnancy outcomes, certain malignancies, neurodegenerative diseases, psychological disorders, and autoimmune conditions. These associations are mediated by 3 primary mechanisms: dysbiotic oral biofilms, chronic low-grade systemic inflammation, and the dissemination of periodontal pathogens throughout the body. The pathophysiology involves elevated levels of pro-inflammatory cytokines (including interleukin 6, tumor necrosis factor alpha, and C-reactive protein), impaired immune function, oxidative stress, and molecular mimicry. Periodontal pathogens, particularly *Porphyromonas gingivalis*, are crucial in initiating and sustaining systemic inflammatory responses. Treatment of periodontitis has demonstrated measurable improvements in numerous systemic conditions, emphasizing the clinical significance of these interconnections. Periodontitis should be understood as more than just a localized oral disease; it significantly contributes to the overall systemic inflammatory burden, with implications for general health. An integrated, multidisciplinary approach to prevention, early detection, and comprehensive treatment is vital for optimal patient outcomes. Healthcare providers should acknowledge oral health as an essential element of systemic well-being.

## Introduction

### Background

Periodontitis is a chronic inflammatory disease known to affect the supportive structures of the teeth [1-3]. In addition to its local impacts, such as gingival inflammation, periodontal pocket formation, and alveolar bone loss, periodontitis is strongly associated with systemic inflammation, which leads to various systemic conditions. These include obesity, diabetes mellitus, cardiovascular disease, pregnancy, chronic kidney disease (CKD), respiratory diseases, rheumatoid arthritis, neurodegenerative diseases, malignancy, stress, depression, and autoimmunity [1-7].

The global prevalence of periodontitis underscores its status as a public health issue. Overall, 7.4% [2] to 11.2% [3] of the adult population exhibit severe periodontitis, with a higher prevalence among older generations. The rising prevalence of this condition in tandem with increasing life expectancy, as well as reductions in root caries-related tooth loss, make periodontitis a primary concern given its adverse economic, social, and health system impacts [2]. In 2015, severe periodontitis accounted for an estimated 3.5 million disability-adjusted life years, exceeding the burden of untreated dental caries [8]. Nevertheless, its indirect consequences, such as reduced chewing efficiency, aesthetic compromise, and diminished quality of life, remain underemphasized [8].

The biological mechanisms underpinning these systemic links are multifactorial, including dysbiotic oral biofilms, chronic low-grade inflammation, and the dissemination of periodontal pathogens and their bioproducts throughout the body [1]. These mechanisms trigger an immune reaction that causes additional local tissue damage while mediating systemic inflammatory states, thereby altering the pathophysiology of diseases beyond the oral cavity [6].

However, findings regarding periodontitis and systemic conditions have sometimes been misinterpreted due to a lack of uniformity in study design, inconsistent disease definitions, and small sample sizes [1-8]. With improved definitions of periodontal diseases and global research guidelines, these issues are now being addressed, paving the way for more robust and reproducible studies [4,5,7].

### Objectives

The purpose of this review is to provide an integrative perspective, based on contemporary evidence, on the relationship between periodontitis and systemic diseases. Grounded in epidemiologic data, biological plausibility, and clinical implications, the review underscores the ongoing need for collaborative care strategies that recognize oral health as an integral component of general health and wellness.

## Ethics statement

As this study is a literature review, it did not require institutional review board approval or individual consent.

## Periodontitis and obesity

Obesity is known to be strongly associated with periodontitis [9-13]. Individuals with a body mass index of 30 kg/m^2^ or greater face a significantly elevated risk of periodontal disease [9,11,14-16]. Many studies have found that obesity contributes to periodontitis through systemic inflammation, altered immune function, and dysbiosis of the oral microbiota [9,10,12,13,17-19]. Studies report that central obesity, particularly when defined using the waist-to-hip ratio, significantly increases the likelihood of developing periodontitis [10-12,18]. Various meta-analyses have demonstrated a linear dose-response correlation of adiposity with the risk and severity of periodontal tissue damage [10,18,20,21]. Prospective studies have revealed that individuals with overweight and obesity experience more rapid progression of periodontitis compared to their normal-weight counterparts [14,16,20].

Obesity drives chronic low-grade systemic inflammation, characterized by elevated levels of pro-inflammatory cytokines, including tumor necrosis factor alpha (TNF-α), interleukin (IL)-6, and C-reactive protein (CRP) [11]. These cytokines exacerbate the destruction of periodontal tissues by disturbing the balance between bone resorption and regeneration [22-24]. Dysbiotic changes in the oral microbiome, such as an increase in gram-negative anaerobic bacteria, create an ideal environment for the development of periodontal disease [17,19]. Other factors, such as adipokines, also link obesity to periodontitis [20,23,25]. For example, increased leptin levels mediate inflammation, while low adiponectin levels impair tissue repair and regeneration [26,27]. Insulin resistance associated with obesity further compromises immune cell function, reducing the capacity of the immune system to defend against bacterial infections of the periodontal tissues [25].

### Clinical guidelines

A comprehensive, multidisciplinary approach is necessary to manage periodontal disease in patients with obesity. Effective periodontal treatment, such as scaling and root planing, helps reduce microbial load and inflammation [28]; however, adjunctive anti-inflammatory medications may also be required. Dentists should emphasize the importance of weight management and lifestyle changes—particularly regarding diet and exercise—in decreasing systemic inflammation. Active collaboration with primary physicians and nutritionists is essential for addressing comorbidities. Regular follow-up appointments should ideally occur every 3 to 4 months to evaluate periodontal disease progression and the effectiveness of treatment strategies [29]. Patient education should focus on fostering intrinsic motivation to maintain good oral health and on understanding the interactive relationship between obesity and periodontal health [28,29].

## Periodontitis and diabetes mellitus

Diabetes mellitus and periodontitis have a well-established reciprocal association. Periodontitis is termed the sixth complication of diabetes, and patients with diabetes are about 3 times more likely to develop severe periodontitis as their nondiabetic counterparts [28,30-33]. Hyperglycemia exacerbates periodontal disease by promoting oxidative stress and the formation of advanced glycation end products [34-37]. Conversely, periodontitis worsens glycemic control by increasing the systemic inflammatory load. Longitudinal research indicates that patients with poorly managed diabetes experience more severe periodontal tissue loss and recover more slowly after therapy compared to those without diabetes.

Diabetes accelerates the deterioration of periodontal tissue through several mechanisms [34]. Oxidative stress induced by chronic hyperglycemia results in an overabundance of advanced glycation end products, which attach to receptors on various cells [35]. This interaction triggers the release of pro-inflammatory cytokines, including TNF-α and IL-6, thereby exacerbating both local and systemic inflammation [38]. Furthermore, reduced neutrophil function in patients with diabetes impairs pathogen removal, while elevated CRP levels contribute to delayed wound healing [39,40]. Pathogens such as *Porphyromonas gingivalis* worsen systemic insulin resistance by triggering inflammatory cytokine cascades [41].

### Clinical guidelines

Effective management of periodontal disease in patients with diabetes requires cooperation between dentists and endocrinologists. Individuals with poorly controlled diabetes should undergo periodontal evaluation every 3 months. Non-surgical therapy, including scaling and root planing, improves glycemic control, with reductions in hemoglobin A1c levels of up to 0.4% [28]. During invasive procedures for patients with uncontrolled diabetes, the dentist should monitor blood glucose levels and administer prophylactic antibiotics. A focused educational program can inform patients about the role of oral health in glycemic control. Individualized oral hygiene measures, including antiseptic mouthwashes and interdental cleaning, should be encouraged for all patients. Nutritional counseling and smoking cessation programs also contribute to improved treatment outcomes [42].

## Periodontitis and cardiovascular disease

Epidemiological evidence supports a robust association between periodontitis and cardiovascular disease [43-45]. Severe periodontitis increases the risk of major adverse cardiovascular events, such as myocardial infarction or stroke, by a factor of 1.4 [44]. The primary mechanisms linking these conditions include chronic inflammation, endothelial dysfunction, and microbial dissemination [46,47]. Periodontal pathogens such as *P. gingivalis* have been detected in atherosclerotic plaques, demonstrating the systemic impact of periodontitis [48]. Longitudinal studies indicate that periodontitis accelerates the progression of cardiovascular disease by elevating levels of certain systemic inflammatory markers, such as CRP [49].

Periodontal inflammation provokes a systemic acute-phase response, increasing levels of CRP, IL-6, and TNF-α [49-52]. These inflammatory mediators can act on the endothelium, causing dysfunction and promoting atherogenesis. Lipopolysaccharides from periodontal pathogens, particularly *P. gingivalis*, circulate into the bloodstream, triggering macrophage foam cell formation and rapid plaque development [53-55]. Periodontal infections also promote platelet aggregation, thereby increasing the risk of thrombosis. Dysbiotic changes in the oral microbiota further induce systemic inflammation, creating a feedback loop that aggravates both periodontal and cardiovascular conditions [56].

### Clinical guidelines

The management of patients with cardiovascular disease complicated by periodontal disease may require additional medications and follow-up visits with a cardiologist. The dentist should assess the risk of bleeding for patients on antiplatelet or anticoagulant therapy before performing invasive procedures [57,58]. Non-surgical periodontal therapy, in conjunction with adjunctive anti-inflammatory medication to reduce systemic inflammation, is a promising approach [59]. Regular dental check-ups, typically every 3 to 4 months, are recommended to monitor oral health and mitigate systemic risk factors, avoiding delayed diagnosis of any issues. Patients should be informed of the potential cardiovascular benefits of maintaining good periodontal health. Preventive lifestyle changes, such as smoking cessation and dietary modifications, are crucial for improving treatment outcomes [60].

## Periodontitis and chronic kidney disease

Periodontitis and CKD share a bidirectional relationship. Patients with CKD are predisposed to eburnation due to immune dysfunction; as such, they may experience persistent periodontal inflammation that, in turn, accelerates CKD progression [61-63]. Observational studies have also shown that patients with advanced periodontal disease are at an increased risk of developing further renal impairment [62,64]. Furthermore, periodontal therapy has been linked to improvements in renal parameters, such as serum creatinine and estimated glomerular filtration rate [65].

Chronic systemic inflammation driven by periodontal pathogens, such as *P. gingivalis*, may contribute to the onset of CKD [66]. Elevated concentrations of pro-inflammatory cytokines—including IL-6, TNF-α, and CRP—further amplify endothelial dysfunction and oxidative stress in renal tissues [67,68]. Bacterial pathogens and their endotoxins can enter the bloodstream, creating a pro-inflammatory state that provokes renal damage. Furthermore, uremia in CKD impairs immune responses, exacerbating the adverse effects on both periodontal and kidney health [69].

### Clinical guidelines

Managing periodontal disease in patients with CKD necessitates close collaboration with nephrologists. Periodontal examinations are recommended every 3 months to control microbial load and, consequently, systemic inflammation. Non-surgical periodontal therapies, such as scaling and root planing, have been shown to be effective in reducing systemic inflammatory markers among those with CKD [70-72]. Dentists should avoid prescribing medications that may compromise renal function and must carefully manage bleeding tendencies in patients taking anticoagulants [65,70]. Patient education should emphasize proper oral hygiene and the interrelationship between periodontal and kidney health. Nutritional counseling for these patients may also include recommendations to reduce sodium and phosphate intake.

## Periodontitis and inflammatory bowel disease

Epidemiological evidence suggests a strong association between inflammatory bowel disease (IBD), including Crohn’s disease and ulcerative colitis, and periodontitis [73]. Patients with IBD exhibit a significantly increased frequency and severity of periodontitis, characterized by greater clinical attachment loss and deeper periodontal pockets [74,75]. Both conditions share common inflammatory pathways, with elevated levels of pro-inflammatory cytokines—namely IL-6, IL-1β, and TNF-α—contributing significantly to tissue destruction at both systemic and local levels [76,77]. Microbiota dysbiosis also plays a major role; periodontitis is marked by an overgrowth of pathogenic bacteria, such as *P. gingivalis* and *Fusobacterium nucleatum*, which may translocate to the gut and exacerbate IBD symptoms [78]. In addition, shared genetic predispositions, including polymorphisms in *IL23R* and *NOD2*, support a common immunological basis for these conditions [79].

### Clinical guidelines

Given this bidirectional interaction, the dental regimen for patients with IBD should include routine periodontal examinations, oral hygiene instruction, and non-invasive periodontal therapy to reduce bacterial load [73,80]. During active IBD flares, extreme caution is advised when scheduling dental treatments. Non-steroidal anti-inflammatory drugs, which may aggravate gut inflammation, should be avoided. Due to the immunosuppressive state, prophylactic antibiotics may be considered [81-83]. Close collaboration between dentists and gastroenterologists is essential to enhance periodontal health and, when possible, reduce systemic inflammation to improve IBD management [84].

## Periodontitis and rheumatoid arthritis

Periodontitis and rheumatoid arthritis share common inflammatory pathways and genetic predispositions, which underpin the relationship between these conditions. Studies have shown that individuals with rheumatoid arthritis are nearly twice as likely to develop periodontitis compared to the general population [85]. This is a reciprocal association, as periodontitis exacerbates systemic inflammation in rheumatoid arthritis, potentially worsening joint symptoms [86,87]. Moreover, observational studies have demonstrated that untreated periodontitis is associated with higher disease activity scores in patients with rheumatoid arthritis, highlighting the impact of local and systemic oral inflammation [86].

The pathogenic link between periodontitis and rheumatoid arthritis primarily involves immune dysregulation driven by *P. gingivalis*, a major periodontal pathogen. This organism produces peptidylarginine deiminase, an enzyme that catalyzes the citrullination of proteins—a hallmark of the pathogenesis of rheumatoid arthritis [88,89]. The citrullination process results in the generation of anti-citrullinated protein antibodies, which promote joint inflammation [90]. Elevated levels of inflammatory cytokines, such as TNF-α, IL-6, and IL-17, are common to both periodontitis and rheumatoid arthritis, contributing to systemic and local tissue destruction [91,92]. Additionally, dysbiosis of the oral microbiome perpetuates inflammatory cycles, creating a vicious feedback loop that increases the severity of both diseases [93].

### Clinical guidelines

Effective management of periodontitis in patients with rheumatoid arthritis requires a multidisciplinary approach. Collaboration between periodontal and rheumatology teams is essential to concurrently address systemic and oral inflammation. Follow-up periodontal examinations should be performed every 3 months. Evidence indicates that non-surgical periodontal therapy, including scaling and root planing, reduces systemic inflammatory markers and improves rheumatoid arthritis symptoms. In severe cases, adjunctive therapies—such as anti-inflammatory or antibiotic treatments—may be considered [94,95]. Patients should receive oral hygiene instructions, including proper techniques for brushing, flossing, the use of interdental brushes, and chlorhexidine rinses. Given that smoking is a known aggravating factor for both rheumatoid arthritis and periodontitis, smoking cessation should be strongly encouraged [96]. Nutritional counseling aimed at avoiding inflammatory food triggers may further improve overall health outcomes for these patients.

## Periodontitis and respiratory diseases

Evidence suggests that periodontitis may play a role in the evolution and exacerbation of respiratory diseases such as pneumonia, chronic obstructive pulmonary disease (COPD), and asthma [97]. Strong evidence indicates that aspiration of oral contents into the respiratory tract can initiate or worsen respiratory tract infections [98,99]. Severe periodontitis is associated with an increased prevalence of pneumonia, particularly among hospitalized and ventilated patients. Studies have also reported a higher tendency for the development of COPD in individuals with periodontitis, with inflammatory markers and microbial load serving as significant mediators [98,100].

The translocation of oral pathogens, including bacteria such as *P. gingivalis* and *F. nucleatum*, into the respiratory tract is a key factor in the pathogenesis of periodontitis-related respiratory diseases [101,102]. These pathogens stimulate local and systemic inflammation, triggering the release of pro-inflammatory cytokines such as IL-1β and TNF-α and thereby aggravating airway inflammation and tissue damage. The dysbiosis observed in the oral cavity and respiratory tract is driven by the uncomplicated colonization of these bacteria. Toxins associated with the periodontium serve as markers of systemic inflammation, weakening the respiratory system’s immune defense against pathogens and increasing the risk of infection [101,102].

### Clinical guidelines

Managing periodontal health in patients with respiratory diseases requires a preventive approach. Regular dental checkups, professional cleanings, and improved oral hygiene practices are essential to minimize the risk of aspiration-related infections. The use of antimicrobial mouthwashes and effective plaque control techniques can significantly reduce the bacterial load in the oral cavity [103]. Collaboration with pulmonologists is recommended for patients with severe respiratory conditions, particularly those who are immunocompromised or on ventilatory support [103]. Patient education should emphasize the importance of maintaining oral health, including smoking cessation, to improve respiratory outcomes and overall quality of life [104].

## Periodontitis and adverse pregnancy outcomes

Periodontitis has been shown to be significantly correlated with adverse pregnancy outcomes, including preterm birth, low birth weight, and preeclampsia [105,106]. In pregnant women with untreated periodontal disease, the risk of such outcomes is about 1.5 times greater than in those without periodontal disease [107]. Periodontal infection is thought to alter fetal intrauterine development through systemic inflammation and the dissemination of microflora from periodontal pockets. Meta-analyses have confirmed the correlation between maternal periodontal disease and preterm birth, particularly among women with severe periodontitis [107]. In addition, elevated levels of inflammatory biomarkers such as IL-6 and CRP in mothers with periodontitis are linked to impaired placental function [108].

The underlying pathophysiology involves both local and systemic inflammatory mechanisms. Oral pathogens, such as *F. nucleatum*, can migrate from the periodontal tissues to the placenta, triggering an immune response that ultimately destabilizes placental integrity [109]. Elevated levels of cytokines, including IL-1β, TNF-α, and prostaglandins, can provoke uterine contractions, leading to preterm birth [110]. Chronic periodontal inflammation potentiates oxidative stress and endothelial dysfunction, jeopardizing fetal nutritional supply and growth [111,112]. Dysbiosis of the maternal oral microbiota further increases systemic inflammation, initiating a cascade of adverse pregnancy events [113].

### Clinical guidelines

Managing periodontal disease in pregnant women requires adherence to specific strategies, as improper management may lead to systemic inflammation. Non-surgical periodontal therapies such as scaling and root planing can be safely performed during the second trimester [114,115]. Regular oral examinations, along the use of antimicrobial mouthwashes and other oral hygiene methods, are consistently recommended for high-risk pregnancies [115-117]. Pregnant women should be informed of the maternal and fetal complications associated with untreated periodontal disease. Obstetricians should collaborate closely for high-risk pregnancies to ensure coordinated healthcare. Moreover, prenatal care may be optimized by incorporating targeted nutritional guidance and smoking cessation support, promoting both maternal and fetal health.

## Periodontitis and malignancy

Periodontitis has been implicated as a precipitating factor for various malignancies, especially oral, pancreatic, and colorectal cancers [118-120]. Chronic inflammation, induced by persistent infection and immune dysregulation, is the primary factor linking periodontitis to tumor development and progression [121]. Certain pathogens, such as *F. nucleatum*, have been shown to play a role in the etiology of colorectal cancer, modulating the tumor microenvironment and stimulating metastasis [122,123].

The connection between periodontitis and malignancy occurs through both direct and indirect mechanisms. The sustained inflammation in periodontal tissues leads to the systemic release of pro-inflammatory cytokines, such as IL-6 and TNF-α, which, in turn, promote angiogenesis and enable immune evasion during tumor growth [124,125]. Oncogenic signaling pathways are activated when oral pathogens, including *F. nucleatum*, adhere to epithelial cells, thereby encouraging cell proliferation and survival [123]. Furthermore, dysbiosis of the oral microbiome fosters additional systemic inflammation that supports carcinogenesis. Increased oxidative stress associated with chronic gingival infections also contributes to DNA damage, potentially heightening the risk of malignant transformation [126,127].

### Clinical guidelines

For patients with cancer or at risk of cancer, intervention aimed at chemoprevention should incorporate maintenance of periodontal health [128]. Periodontal examinations and cleanings should be performed regularly to reduce systemic inflammation and microbial load [129]. Collaboration with oncologists is necessary to develop an effective dental care protocol, especially for patients undergoing chemotherapy and radiation therapy, whose side effects impact oral health [119]. Specifically, dentists should educate patients on the benefits of maintaining proper oral hygiene—such as regular brushing—and the use of topical measures, including fluoride applications and antimicrobial rinses [130,131]. Nutritional counseling may also help bolster immune support and manage systemic inflammation.

## Periodontitis and neurodegenerative diseases

The mechanisms linking periodontitis with neurodegenerative diseases, such as Alzheimer’s disease and Parkinson’s disease, include both systemic inflammation and direct microbial invasion [132-135]. Periodontal pathogens, particularly *P. gingivalis*, produce virulent factors—specifically, gingipains—that compromise the integrity of the blood-brain barrier, allowing bacteria and inflammatory mediators to enter the central nervous system [136]. This process triggers microglial activation and the release of pro-inflammatory cytokines, such as TNF-α and IL-1β, which further contribute to neuronal damage [137,138]. Additionally, *P. gingivalis* has been shown to induce the deposition of amyloid-β plaques and the phosphorylation of tau proteins, both hallmarks of Alzheimer’s disease [139]. Systemic inflammation caused by chronic periodontitis may further augment oxidative stress and neuroinflammation, thereby accelerating neurodegeneration [140].

### Clinical guidelines

For patients at risk of developing neurodegenerative diseases, as well as those already diagnosed, an ongoing, preventive approach to managing periodontitis is recommended. Regular dental appointments and periodic cleanings should be instituted to minimize the microbial burden and reduce systemic inflammation [141]. Caregivers should be involved to help ensure adherence to proper oral health practices, such as brushing with fluoride toothpaste and using an antimicrobial mouthwash. Collaboration with neurologists is recommended to monitor the interplay between oral and cognitive health. Dentists should educate patients on the importance of maintaining oral health for overall neurological function. For individuals in the advanced stages of neurodegenerative disease, care plans should be personalized to account for physical and cognitive limitations [142].

## Periodontitis, stress, and depression

Stress and depression are strongly correlated with periodontitis through both behavioral and physiological mechanisms. Studies indicate that individuals experiencing chronic stress or depression may require more extensive periodontal treatment, with odds ratios reaching approximately 1.5 compared to those without these conditions [143-145]. Lifestyle factors common among stressed or depressed individuals, such as poor oral hygiene, smoking, and unfavorable dietary practices, can exacerbate the progression of periodontal disease [144,146]. Additionally, depression heightens systemic inflammation, contributing to periodontal tissue breakdown [147]. Stress-related hormones, particularly cortisol, interfere with the immune response by suppressing the activity of immune cells that target periodontal pathogens, which can lead to increased bacterial proliferation and inflammation in periodontal tissues [148]. In conjunction with depression, stress elevates the release of pro-inflammatory cytokines such as IL-6 and TNF-α, further worsening tissue destruction and bone resorption [148].

### Clinical guidelines

Managing periodontal disease in patients experiencing stress or depression requires a holistic approach that addresses both psychological and oral health [149,150]. Dentists should collaborate with mental health professionals to provide integrated care. Regular periodontal evaluations and cleanings are recommended to control bacterial load and reduce inflammation. To improve overall health outcomes, stress relief options such as mindfulness, counseling, or cognitive-behavioral therapy should be suggested. Dentists should educate patients about the importance of maintaining good oral hygiene and the bidirectional relationship of stress and depression with periodontal health. In addition, treatments may include the use of antimicrobial mouthwashes and anti-inflammatory medications to help control inflammation. Furthermore, smoking cessation and nutritional counseling to support immune function should be key components of the treatment regimen [151,152].

## Periodontitis and autoimmunity

Autoimmune diseases such as systemic lupus erythematosus, diabetes mellitus type 1, and rheumatoid arthritis share inflammatory pathways and dysregulated immune responses with periodontitis. In these conditions, periodontal attachment loss is often particularly severe. Meta-analyses have revealed that systemic lupus erythematosus significantly increases the risk of periodontitis compared to the general population [153,154]. These conditions are characterized by elevated inflammatory markers, such as CRP, which link systemic inflammation with the progression of oral disease. However, apart from studies on rheumatoid arthritis, there is a marked deficiency of well-designed studies investigating the correlation between periodontitis and other autoimmune diseases, largely due to methodological weaknesses—particularly the inconsistent use of clinical indices. Thus, the conclusions that can be drawn at this time are limited and should be interpreted with discretion [155-158]. *P. gingivalis*-induced molecular mimicry and immune dysregulation exacerbate the autoimmune response by elevating IL-17 levels, which in turn causes further destruction of periodontal tissues [159]. In the production of autoantibodies, a process known as citrullination, *P. gingivalis* has been shown to contribute to several autoimmune diseases, especially rheumatoid arthritis. Dysbiosis of the oral microbiome enhances systemic immune activation and further impairs tissue repair mechanisms. Moreover, periodontal pathogens stimulate dendritic cell maturation and the release of pro-inflammatory cytokines, thereby intensifying autoimmune activity and tissue destruction [160].

### Clinical guidelines

Patients with autoimmune diseases require multidisciplinary care to effectively manage both systemic and periodontal inflammation. Coordination between dental professionals and rheumatologists can facilitate favorable treatment outcomes. Periodontal debridement should ideally be performed at least every 3 months, depending on the severity of the condition, to reduce bacterial load and inflammation. In addition, anti-inflammatory and immunomodulatory therapies tailored to the patient’s systemic condition may positively impact periodontal outcomes [161]. Dentists must carefully evaluate and consider the potential oral side effects of systemic treatments, such as dry mouth caused by immunosuppressive therapies; in such cases, adjunctive therapies like topical fluorides and artificial saliva support may be necessary [161-163]. For patients receiving biologic or high-dose immunosuppressive therapies, medical clearance may be required prior to dental treatment [162].

## Conclusion

Periodontitis is a chronic inflammatory condition with significant implications for systemic health. It is more than simply a localized oral disease, as evidenced by its established associations with systemic conditions such as diabetes, cardiovascular disease, adverse pregnancy outcomes, respiratory disorders, autoimmune diseases, and neurodegenerative diseases. These links are mediated by dysbiotic biofilms, systemic inflammation, and the dissemination of bacterial components and inflammatory mediators into the circulation—mechanisms implicated in the pathogenesis of numerous systemic diseases.

Despite extensive research on periodontitis, historical impediments such as varying definitions of the disease, differences in study design, and small sample sizes have limited the comparability of findings. However, recent advances in classification systems and evidence-based research guidelines have substantially improved the quality and interpretability of periodontal studies, enabling researchers to better understand the systemic implications.

This review underlines the need for an integrated approach to the management of periodontitis. Collaborative care across dental and medical disciplines can address the systemic effects of periodontitis and oral diseases, improving both oral and general health outcomes. Given the rising prevalence of periodontitis due to aging populations, increased life expectancy, and lifestyle issues, such an effort is especially timely.

In addressing periodontitis and systemic disease, priority should be given to prevention, early detection, and comprehensive treatment within an integrated model of care. Interdisciplinary networks should be established and strengthened to emphasize evidence-based interventions for individuals with periodontitis and those at risk, ultimately enhancing intervention options. By considering oral health as an integral component of systemic well-being, healthcare providers can be better positioned to improve quality of life and mitigate the overall impact of this widespread disease.
